# Exploring the Impact of miR‐34b‐5p on BRD4 Gene Expression in Triple‐Negative Breast Cancer Cells

**DOI:** 10.1155/bmri/7538031

**Published:** 2025-08-28

**Authors:** Mohannad Abdulameer Ibrahim, Hanieh Jalali, Mahnaz Azarnia

**Affiliations:** ^1^ Department of Animal Biology, Faculty of Biological Sciences, Kharazmi University, Tehran, Iran, khu.ac.ir

**Keywords:** apoptosis, breast cancer, bromodomain-containing proteins, microRNA

## Abstract

The miR‐34 family is recognized for its crucial role as tumor suppressors, particularly through its interactions with oncogenic regulators such as Tumor Protein 53 (TP53). Bromodomain‐containing Protein 4 (BRD4) functions as a transcriptional regulator that enhances the expression of oncogenes. In triple‐negative breast cancer (TNBC), BRD4 is often found to be overexpressed and linked to poor clinical outcomes. This study is aimed at exploring the impact of miR‐34b on the sensitivity of TNBC cells to BRD4 inhibition, which may also affect TP53 expression. miR‐34b‐5p mimics and scrambled oligonucleotides were transfected into TP53‐mutant MDA‐MB‐231 and nonmutant MCF‐7 cell lines. The expression levels of miR‐34b, TP53, and BRD4 genes, along with the migration rates and sensitivity to the BRD4‐specific inhibitor JQ1, were compared between the miR‐34b overexpressing cells. The results showed that miR‐34b overexpression led to increased TP53 expression in MDA‐MB‐231 cells, while a reduction was observed in MCF‐7 cells. Consequently, BRD4 expression was significantly elevated in MDA‐MB‐231 cells, resulting in resistance to JQ1. The increase in BRD4 expression also correlated with higher migration rates compared to MCF‐7 cells. In conclusion, miR‐34b may have an oncogenic role by promoting the expression of BRD4 in TNBC cells. This finding aligns with prior reports indicating a negative correlation between miR‐34b levels and survival rates in patients with TNBC. These insights may provide new perspectives on the role of miR‐34b in the development and progression of TNBC.

## 1. Introduction

The miR‐34 family, which comprises miR‐34a, miR‐34b, and miR‐34c, plays a significant role as tumor suppressors in cancer [1]. The large number of target genes under the control of miR‐34s is a testament to their versatility and importance in regulating cellular functions. Specifically, the miR‐34 family interacts with a wide range of oncogenic regulators, such as Tumor Protein p53 (TP53) and neurogenic locus notch homolog Protein 1 (NOTCH1), along with key components of the wingless (Wnt) and transforming growth factor *β* (TGF‐*β*) signaling pathways [2]. These interactions create a complex regulatory network that significantly impacts cellular apoptosis and modulates essential signaling cascades. The potential use of miR‐34 mimics to restore normal function in tumor cells has been considered for therapy, especially in cases where traditional treatments may fail [3]. miR‐34s have been considered in the treatment of breast cancer, where the expression of miR‐34 family members is often downregulated compared to normal breast tissue [4]. This dysregulation contributes to tumorigenesis and progression. Studies indicate that restoring the expression of miR‐34 can inhibit breast tumor growth and enhance sensitivity to chemotherapy. However, reports show a negative correlation between miR‐34b—but not miR‐34a or miR‐34c—and the survival rates of triple‐negative breast cancer (TNBC) patients, indicating that miR‐34b likely plays an oncogenic role in TNBC rather than a tumor‐suppressive one [5]. Also, a cohort study on colon tumors indicated that higher miR‐34b expression correlates with more advanced colon tumors [6]. This finding is contrary to the traditional view of miR‐34 family members as tumor suppressors.

TNBC is a highly aggressive tumor that accounts for 10%–20% of breast cancer cases and lacks targeted treatments [7, 8]. The TP53 gene is found to be mutated in approximately 80% of TNBC cases, which is often associated with more aggressive disease and poorer outcomes [9]. The majority of TP53 mutations occur within the DNA‐binding domain, leading to the production of an inoperative protein that is unable to respond to cellular stress [10, 11]. TP53 mutations can lead to altered gene expression patterns in TNBC; for example, the presence of mutant p53 proteins can enhance the transcriptional activity of c‐MYC, leading to increased oncogenic signaling [12].

Bromodomain‐containing Protein 4 (BRD4), a member of the bromodomain and extraterminal (BET) family, is involved in transcriptional regulation and cell cycle progression [13]. It functions as an epigenetic reader, recognizing acetylated lysine residues on histones and facilitating the recruitment of transcriptional machinery. Recent investigations have demonstrated that BRD4 and TP53 can interact, with BRD4 modulating p53‐dependent transcriptional programs [14]. However, there are reports that TP53 is a direct miR‐34 target in different cancers [15], but the regulatory relationships between TP53, BRD4, and miR‐34b are not fully elucidated in TNBC. Understanding the interplay between these factors is essential for developing targeted cancer therapies and enhancing our knowledge of cellular regulatory mechanisms. In this study, we aimed to investigate whether the overexpression of miR‐34b influences the sensitivity of TNBC cells to BRD4 inhibition, potentially by modulating TP53 expression. To achieve this, we designed experiments to overexpress miR‐34b in both MDA‐MB‐231 and MCF‐7 cells. We assessed the expression levels of BRD4 and TP53 following miR‐34b overexpression and evaluated the sensitivity of miR‐34b‐overexpressing cells to JQ1, a small molecule with BRD4 inhibitory function.

## 2. Materials and Method

### 2.1. Ethics Statements

This study was conducted in accordance with the research ethics code IR.KHU.REC.1403.001, which was approved by the Research Ethics Committee of Kharazmi University.

### 2.2. Cell Culture

MDA‐MB‐231 and MCF‐7, human breast cancer cell lines, were purchased from the Pasteur Institute (Iran) and cultured in T75‐cm^2^ flasks in Dulbecco’s modified Eagle’s medium (Gibco, United Kingdom), supplemented with 5% fetal bovine serum (FBS; Gibco, United Kingdom) and 1% penicillin–streptomycin solution (Gibco, United Kingdom). The cells were maintained at 37°C in a humidified 5% CO_2_ atmosphere. Upon reaching a cell density of approximately 80%, the cells underwent transfection.

### 2.3. Transfection of Cells With miR‐34b‐5p Mimics and Scrambled Oligonucleotides

miR‐34b‐5p mimic with sequence 5 ^′^‐UAGGCAGUGUCAUUAGCUGAUUG‐3 ^′^ and scrambled oligonucleotide were purchased from miRas Biotech Co. (Iran) and were dissolved in RNase DEPC water and stored at −20°C. For cell transfection, both cell lines underwent transfection with oligonucleotides utilizing DNA‐fectamine (Bio Basic, Canada) in accordance with the established protocol. Briefly, cells were plated at a density as shown in Table 1. On the subsequent day, the cells were transfected with the oligonucleotides at a ratio reported in Table 1, in a serum‐ and antibiotic‐free medium. Following a 14–16‐h incubation period, the growth media were replaced with fresh media, and the cells in the initial plate were incubated under appropriate experimental conditions for an additional time. To verify the efficacy of the transfection, cells were labeled with a delivery‐check fluorescein amidite oligonucleotide (miRas Biotech Co., Iran), and the proportion of labeled cells was assessed using a flow cytometer (BD FACSLyric, United States).

**Table 1:** The specific quantities of reagents utilized for cell transfection.

**Culture vessels**	**Cell (×10** ^**6**^ **)**	**miR-34b-5p mimic (*μ*g)**	**Scrambled oligonucleotides (*μ*g)**	**DNA-fectamine (*μ*L)**
6 well	1.2	3	3	10
12 well	0.30	1.6	1.6	4
24 well	0.14	0.8	0.8	2
96 well	0.01	0.2	0.2	0.5

### 2.4. Determining the Cell Viability Using 3‐(4,5‐Dimethylthiazol‐2‐yl)‐2,5‐Diphenyltetrazolium Bromide (MTT) Assay

The viability of cells after transfection or treatment was assessed using the MTT assay. Briefly, 16 h after transfection, the transfection media were replaced with culture media, and the cells were incubated for 48 h. For JQ1 treatment, cells were seeded in each well of 96‐well plates and exposed to 1, 5, 10, and 15 *μ*M JQ1 for 24 h. After the treatment period, the media were discarded, and 10 *μ*L of MTT solution (5 mg/mL; Sigma‐Aldrich, United Kingdom) was added to each well, followed by an additional 4 h incubation at 37°C in the dark. The medium was then removed, and 100 *μ*L of dimethyl sulfoxide (DMSO; Merck, Germany) was added to each well. The optical density of the samples was measured at a wavelength of 570 nm using a microplate reader (Bio‐Rad, United States). The percentage of viable cells was calculated by comparing the number of viable cells to that of the control sample.

### 2.5. Determining the Expression of miR‐34b‐5p Post Cell Transfection

The quantitative reverse transcription–polymerase chain reaction (qRT‐PCR) technique was employed to evaluate the expression levels of miR‐34b‐5p in the transfected cells, as well as nontransfected cells as endogenous control. Specifically, MDA‐MB‐231 and MCF‐7 cells were transfected as described earlier. Forty‐eight hours posttransfection, the cells were washed with RNase‐free PBS, and total cellular RNA was extracted using TRIzol reagent (Thermo Fisher Scientific, United States). The stem‐loop qPCR approach was used for the identification and quantification of miRNA in the transfected cells. Briefly, the stem‐loop probe “stem‐loop Seq” was used to specifically capture miR‐34b‐5p and perform reverse transcription with RevertAid reverse transcriptase (Thermo Fisher Scientific, United States). The qRT‐PCR was performed using SYBR Green PCR Master Mix (Thermo Fisher Scientific, United States), adhering to the manufacturer’s guidelines. The qRT‐PCR cycling conditions were as follows: 94°C for 30 s, 60°C for 30 s, and 72°C for 30 s, with a total of 40 cycles. The reactions were processed and analyzed using the Rotor‐Gene Q Real‐Time PCR System (QIAGEN, Germany). U6 was used as an internal reference gene, and miR‐34b expression levels in transfected cells were reported relative to those in nontransfected cells. The primer sequences for the target and reference genes are detailed in Table 2.

**Table 2:** Sequence of primers in qRT‐PCR analysis.

**Name**	**Sequences (5** ^′^ **-3** ^′^ **)**
Stem‐loop Seq	GTCGTATCCAGTGCAGGGTCCGAGGTATTCGCACTGGATACGACCAATCAG
U6 snRNA‐F	GTGCTCGCTTCGGCAGC
U6 snRNA‐R	TATCCAGTGCAGGGTCCGA
miR‐34b‐5p‐F	AAGCGACCTAGGCAGTGTCATT
miR‐34b‐5p‐R	GTCGTATCCAGTGCAGGGTCC
BRD4‐F	CGCTATGTCACCTCCTGTTTGC
BRD4‐R	ACTCTGAGGACGAGAAGCCCTT
TP53‐F	CCTCAGCATCTTATCCGAGTGG
TP53‐R	TGGATGGTGGTACAGTCAGAGC
c‐MYC‐F	CCTGGTGCTCCATGAGGAGAC
c‐MYC‐R	CAGACTCTGACCTTTTGCCAGG
B_2_M‐F	GACCACTTACGTTCATTGACTCC
B_2_M‐R	CAGGGTTTCATCATACAGCCAT

### 2.6. Examining the Expression of BRD4, TP53, and c‐MYC Genes Using Real‐Time PCR

The qRT‐PCR technique will be used to assess the expression levels of the BRD4, TP53, and c‐MYC genes in the MDA‐MB‐231 cell line, as well as the MCF‐7 cell line, following the 48 h post‐transfection. Nontransfected cells were used as controls in all steps. Briefly, the total RNA was extracted from cells and converted to cDNA; then, the qRT‐PCR was performed according to the protocol described in Section 2.4. The relative expression levels of the target genes were determined using the 2^−*ΔΔ*CT^ method, with B_2_M as the reference gene. Gene expression levels in transfected cells were reported relative to those in nontransfected cells. The primer sequences for the targeted and reference genes are provided in Table 2.

### 2.7. Annexin V Assay

Apoptosis was assessed using an annexin V‐FITC apoptosis detection kit (MabTag, Germany). MDA‐MB‐231 and MCF‐7 cell lines were cultured in a 6‐well plate and allowed to incubate for 24 h before transfection or treatment. Following this incubation period, transfection or treatment was carried out as described. After 48 h, the cells were harvested via trypsinization and resuspended in 90 *μ*L of binding buffer. The resulting cell suspensions were incubated with 5 *μ*L of annexin V‐FITC and 5 *μ*L of propidium iodide (PI) for 20 min at room temperature, protected from light. Flow cytometry (BD FACSLyric, United States) was then used to analyze the cells.

### 2.8. Cell Migration Assay

MDA‐MB‐231 and MCF‐7 cell lines transfected with miR‐34b were cultured in 24‐well plates. Subsequently, a sterile tip was used to apply pressure to the cell monolayer, creating a clear line. The cells were then washed three times with PBS to remove detached cells. Cell migration was monitored over 48‐h period, during which photographs of each well within a defined area were taken using a camera adapted for use with an inverted microscope.

### 2.9. Statistical Analysis

All experiments were performed in triplicate or more. Statistical analyses were conducted using GraphPad Prism 9.5.1 (GraphPad Software Inc., La Jolla, California, United States). Two‐ or one‐way ANOVA with Tukey’s post hoc test was applied, considering *p* < 0.05 as statistically significant. REST 2.0.13 software was used for RT‐PCR data analysis. The migration area was quantified using ImageJ software Version 1.52. The data of flow cytometry was quantified using FlowJo software Version 10.5.3.

## 3. Results

### 3.1. Expression of miR‐34b in Transfected Cells

Analysis of the green fluorescent protein expression using flow cytometry revealed a transfection efficiency of over 90% (Figure 1a,b). Furthermore, the assessment of miR‐34b expression in the transfected cells indicated that miR‐34b levels were significantly elevated in both transfected cell lines compared to nontransfected cells. In contrast, transfection with an oligonucleotide lacking the expression sequence had no impact on miR‐34b expression in either cell line (Figure 1c).

Figure 1Assessment of transfection efficiency and miR‐34b expression in transfected cells. (a) Representative image of green fluorescent emission from fluorescein oligonucleotide‐transfected cells. (b) Percentage of cells transfected with fluorescein oligonucleotide. (c) Expression level of miR‐34b in transfected cells.

**Figure:**
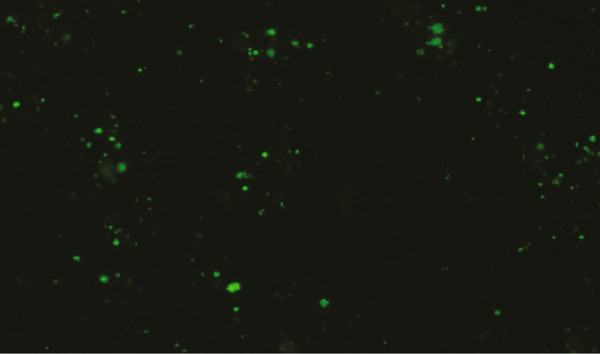
(a)

**Figure:**
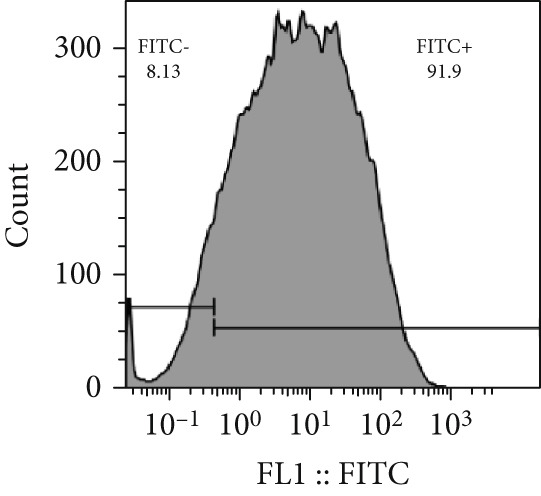
(b)

**Figure:**
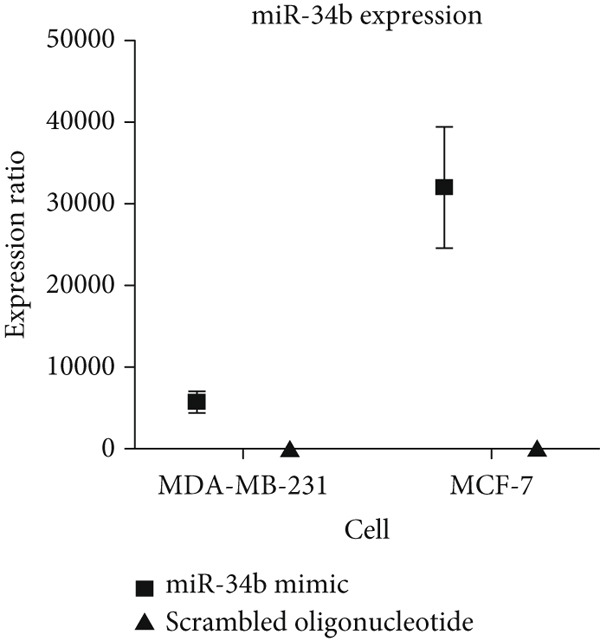
(c)

### 3.2. Cell Viability in miR‐34b‐Transfected Cells

The results of the MTT assay and annexin analysis indicated that miR‐34b transfection led to a decrease in cell survival and an increase in cell death in both transfected cell lines. However, the reduction in cell population was significantly pronounced in the MCF‐7 line compared to the MDA‐MB‐231 line (Figure 2a). Specifically, 48 h after the introduction of the miR‐34b sequence, approximately 60% of the MCF‐7 cells underwent apoptosis, while about 16% of the MDA‐MB‐231 cells underwent apoptosis (Figures 2b, 2c, 2d, and 2e).

Figure 2Cell viability following miR‐34 transfection. (a) Percentage of viable cells 48 h posttransfection determined by MTT assay. (b) The percentage of viable and dead cells following miR‐34b transfection to MDA‐MB‐231 and MCF‐7 cells quantified using flow cytometry analysis. (c–e) Representative images of flow cytometry analysis showing Q1, necrotic; Q2, preapoptosis; Q3, late apoptosis; and Q4, live cells.

**Figure:**
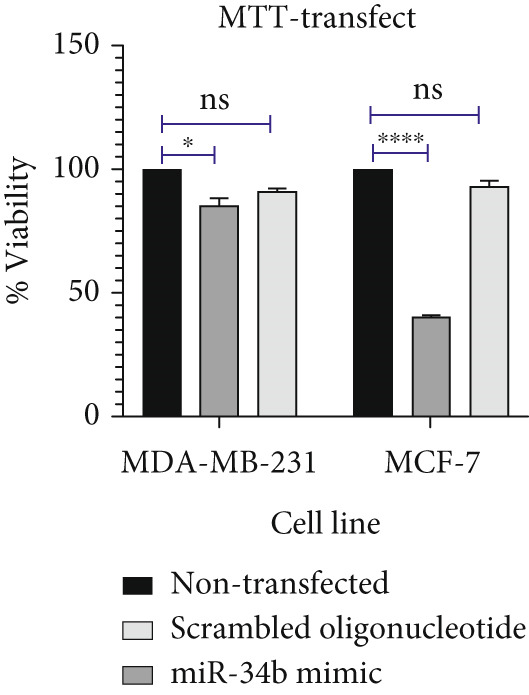
(a)

**Figure:**
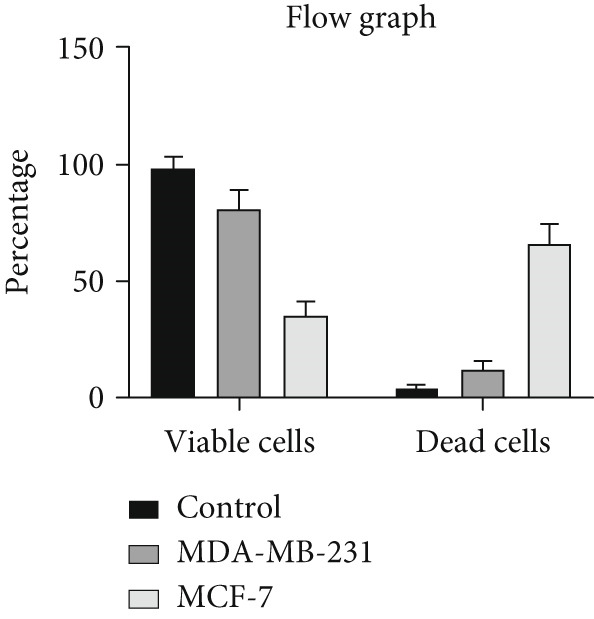
(b)

**Figure:**
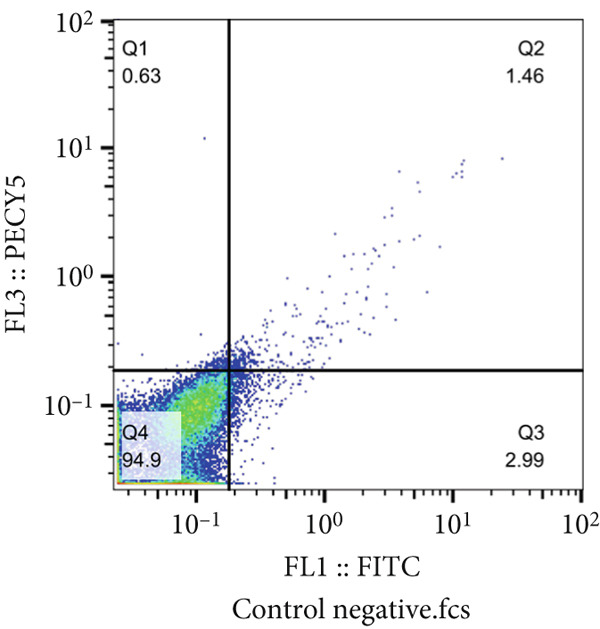
(c)

**Figure:**
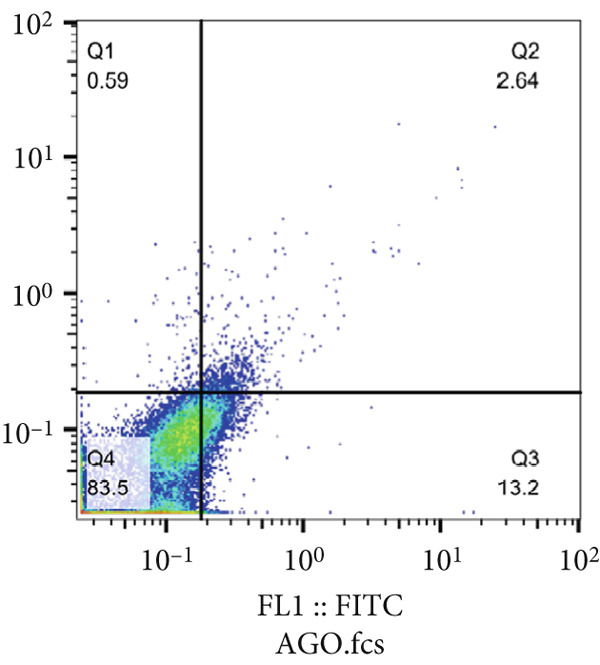
(d)

**Figure:**
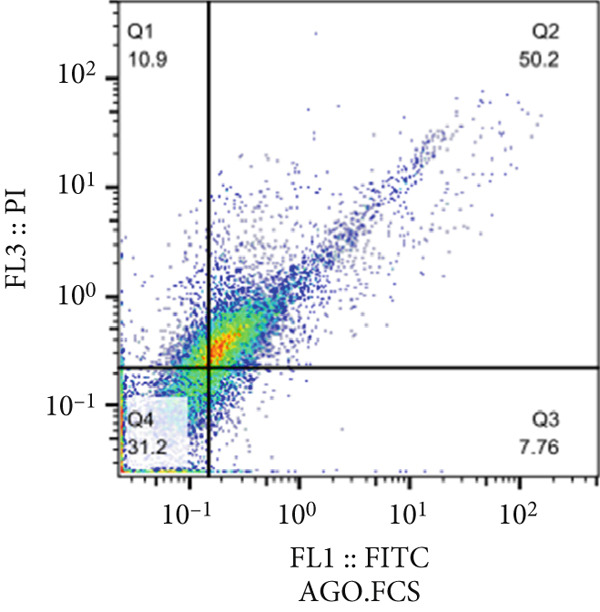
(e)

### 3.3. Expression of BRD4, TP53, and c‐MYC Genes in miR‐34b‐Transfected Cells

**Figure 3:**
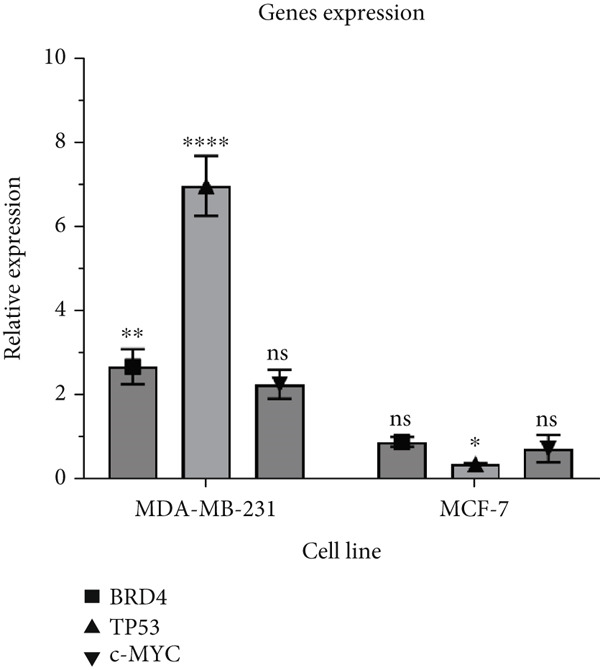
Gene expression following miR‐34b overexpression. Quantitative relative expression of BRD4, TP53, and c‐MYC genes in MDA‐MB‐231 and MCF‐7 cells 48 h after miR‐34b transfection. Data are presented as mean ± SD.  ^∗^ *p* < 0.05;  ^∗∗^ *p* < 0.01;  ^∗∗∗∗^ *p* < 0.0001.

To examine the effects of miR‐34b sequence transfer on the expression of the TP53, BRD4, and c‐MYC genes, qRT‐PCR was performed. The results indicated that in the MDA‐MB‐231 cells, transfection led to a significant increase in the expression of both TP53 and BRD4 genes, while no change was observed in c‐MYC expression. In contrast, in the MCF‐7 cells, TP53 expression was relatively lowered, and there were no changes in the expression levels of BRD4 and c‐MYC (Figure 3).

### 3.4. Resistant to JQ1 Inhibitory Effect in miR‐34b‐Transfected Cells

To determine the inhibitory dose of JQ1, an MTT toxicity assay was conducted, revealing that JQ1 effectively inhibited the growth of MDA‐MB‐231 cells within a concentration range of 1–15 *μ*M (Figure 4a). A concentration of 5 *μ*M was selected for simultaneous treatment with the transfected cells. Analysis of BRD4 gene expression showed that treatment with JQ1 led to a decrease in BRD4 expression in both nontransfected cell lines; however, in transfected MDA‐MB‐231 cells, JQ1 treatment did not result in a significant reduction in BRD4 levels, and BRD4 expression was comparable with control cells. In contrast, in transfected MCF‐7 cells treated with JQ1, BRD4 expression was the same as nontransfected cells, without significant change (Figure 4b,c). Assessment of death type and quantity indicated that while simultaneous treatment with JQ1 significantly increased the number of apoptotic cells in MCF‐7 cells, this effect was not observed in MDA‐MB‐231 cells (Figures 4d, 4e, 4f, 4g, 4h, and 4i).

Figure 4Inhibitory function of JQ1 in miR‐34b‐transfected cells. (a) Determining the inhibitory concentration of JQ1 against MDA‐MB‐231 cells over a 24‐h period. (b, c) Comparison of BRD4 gene expression between miR‐34b‐transfected and nontransfected cells treated with JQ1. (d–g) Apoptosis rate in miR‐34b‐transfected and nontransfected cells treated with JQ1. (h, i) Comparison of the rate of viable and dead cells between miR‐34b‐transfected and nontransfected cells treated with JQ1. Data are presented as mean ± SD; different letters indicate significant difference at *p* < 0.05 level.

**Figure:**
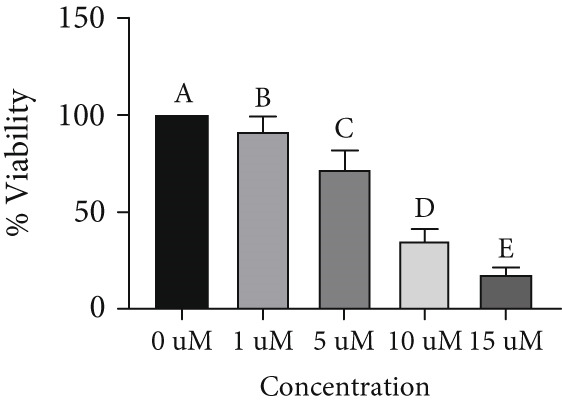
(a)

**Figure:**
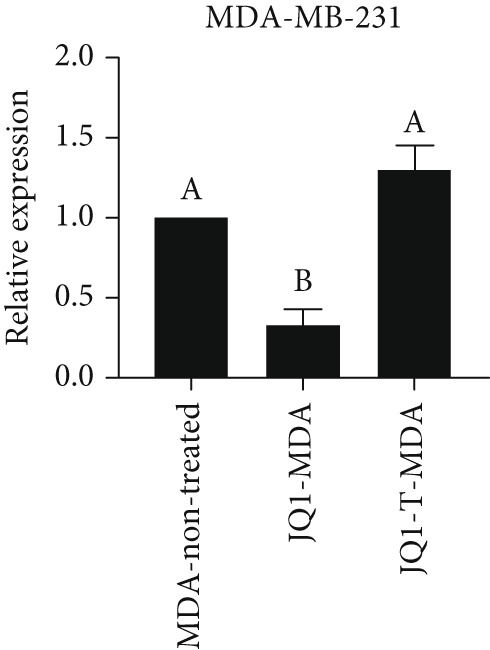
(b)

**Figure:**
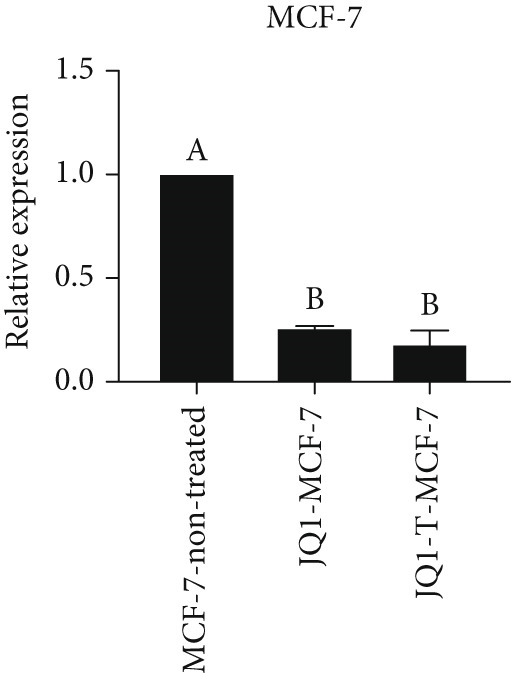
(c)

**Figure:**
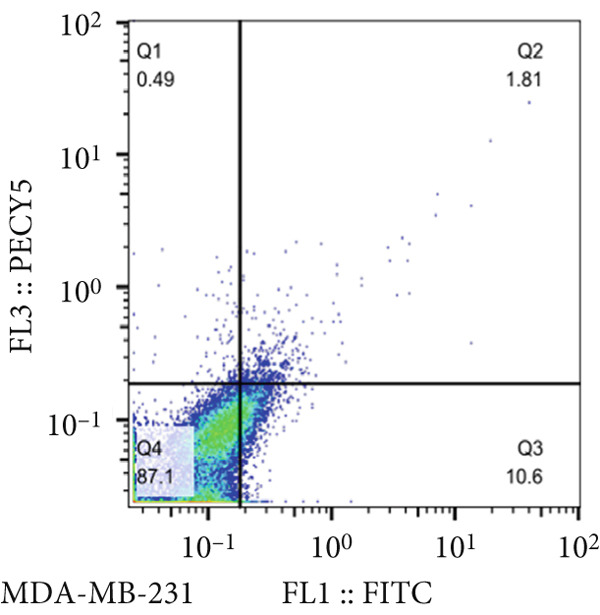
(d)

**Figure:**
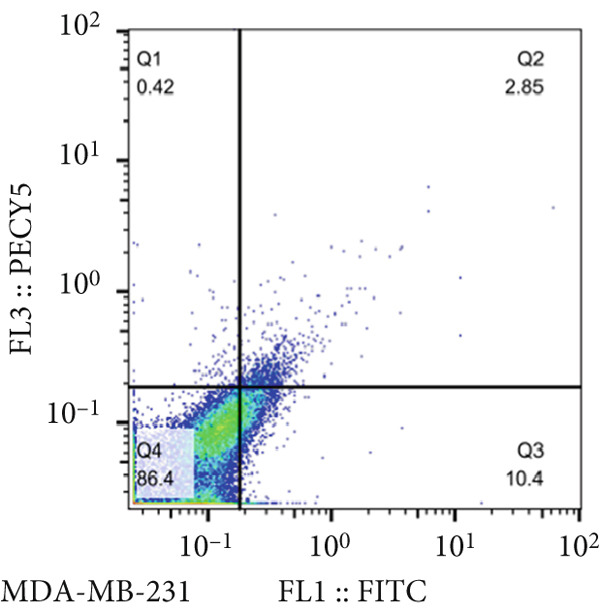
(e)

**Figure:**
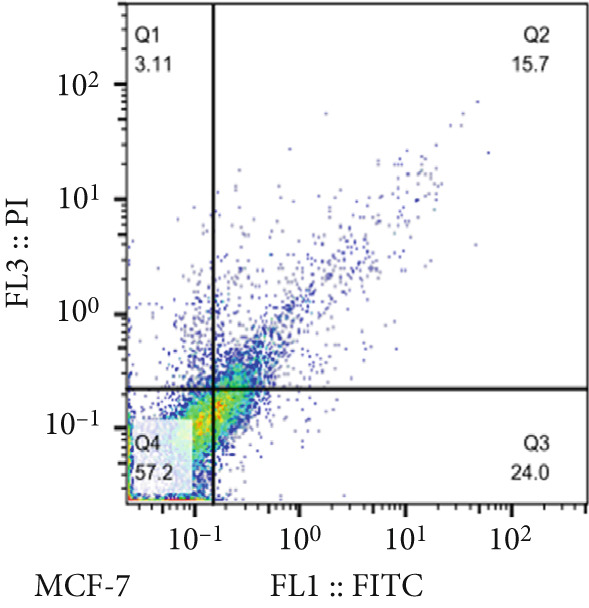
(f)

**Figure:**
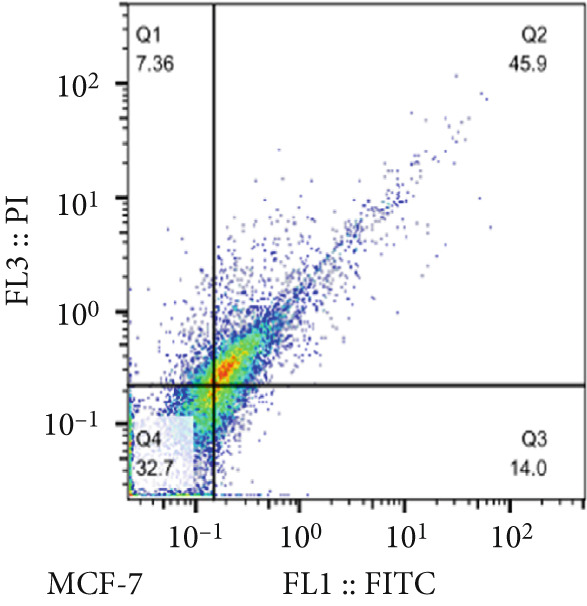
(g)

**Figure:**
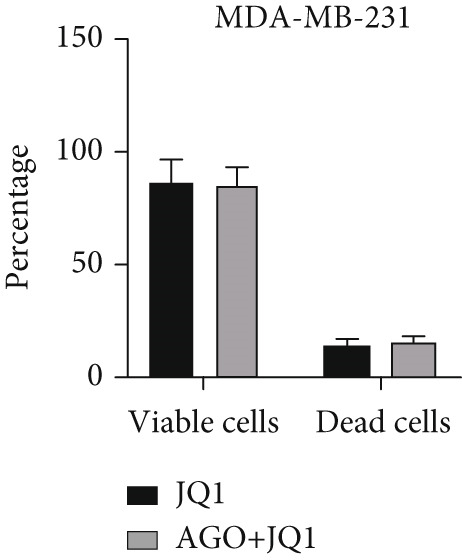
(h)

**Figure:**
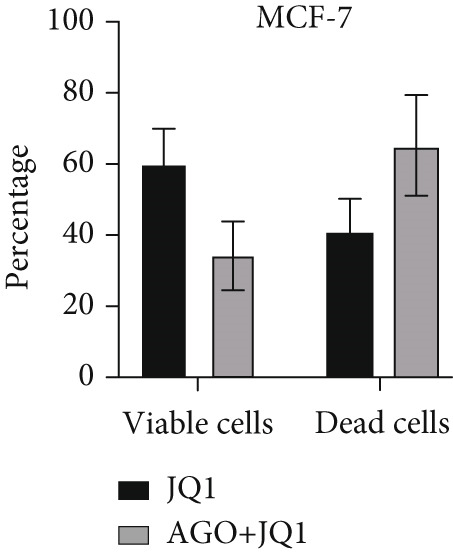
(i)

### 3.5. Metastatic Function of miR‐34b‐Transfected Cells

At 24 h posttransfection, miR‐34b significantly reduced cell migration in both cell lines. By 48 h, the inhibition of migration persisted in MCF‐7 cells; however, in MDA‐MB‐231 cells, this effect was lost as the scratch wound had fully healed. Notably, the combination of JQ1 and miR‐34b (Ago + JQ1) did not produce a greater inhibitory effect on migration of MDA‐MB‐231 cells compared to miR‐34b alone. In contrast, in MCF‐7 cells, this combination enhanced the migration inhibition. At 48 h, while JQ1 alone did not significantly affect migration compared to the control group in MCF‐7 cells, both the Ago + JQ1 and the miR‐34b groups showed a significant reduction in migration relative to the control group (Figure 5).

Figure 5Metastatic function of miR‐34b‐transfected cell. (a) Comparison of cell migration rates in MDA‐MB‐231 cells under various conditions, including miR‐34b transfection (Ago), JQ1 treatment, and the combination of both transfection and treatment (Ago + JQ1). (b) Comparison of cell migration rates in MCF‐7 cells under various conditions, including miR‐34b transfection (Ago), JQ1 treatment, and the combination of both transfection and treatment (Ago + JQ1). The lines represent the diameter of cell‐free areas measured on a micrometer scale (micrometer). Data are presented as mean ± SD.  ^∗^ *p* < 0.05;  ^∗∗^ *p* < 0.01;  ^∗∗∗^ *p* < 0.001;  ^∗∗∗∗^ *p* < 0.0001.

**Figure:**
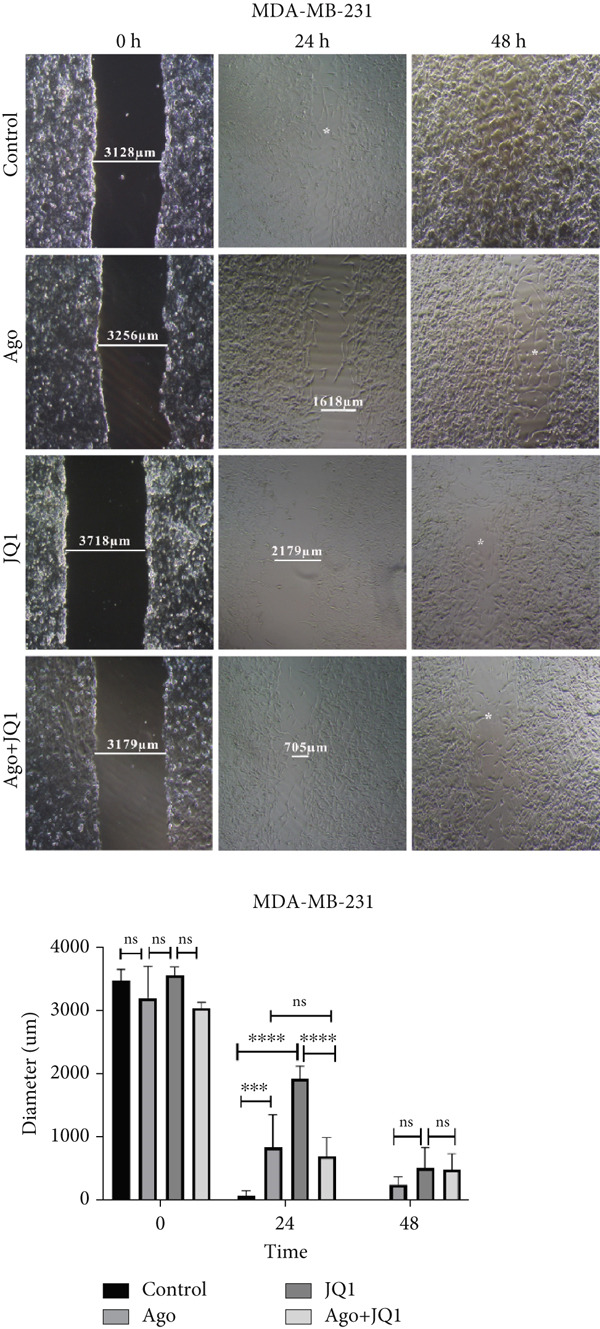
(a)

**Figure:**
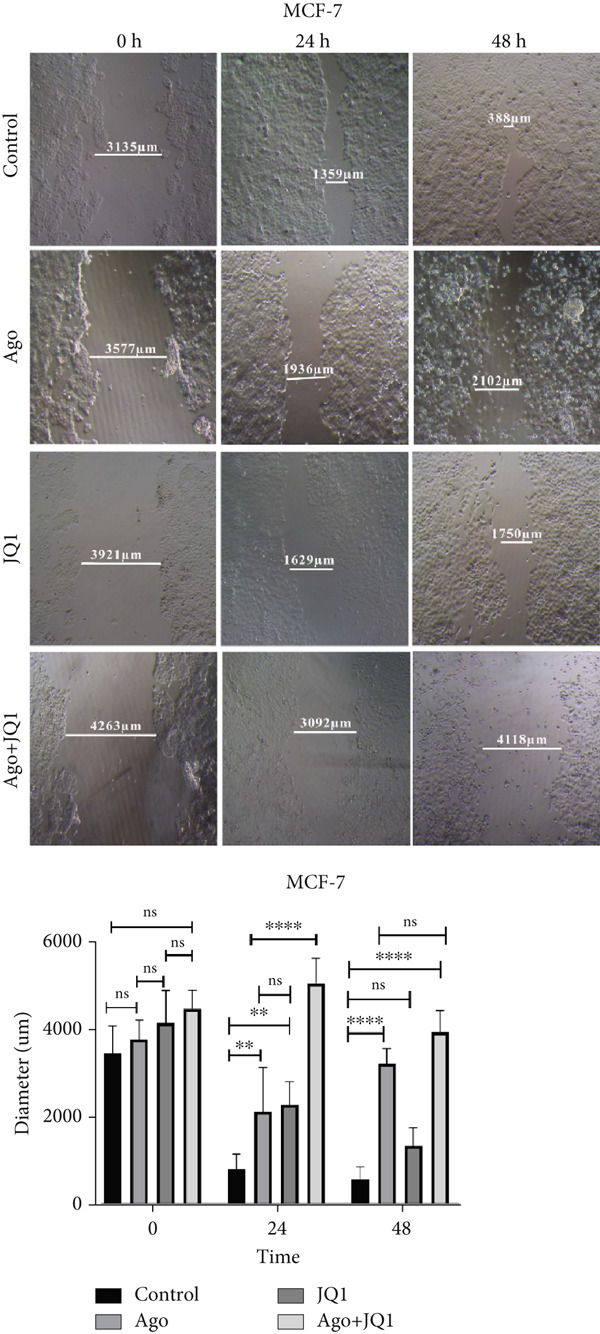
(b)

## 4. Discussion

In this study, we examined the role of miR‐34b in regulating BRD4 expression. Our findings revealed that in MDA‐MB‐231 cells, an increase in miR‐34b expression correlated with elevated BRD4 levels, leading to resistance against JQ1 treatment. Conversely, in the MCF‐7 cell line, while miR‐34b expression rose, BRD4 expression remained unaffected. These results can be understood from several perspectives.

Members of the miR‐34 family are downstream targets of the transcription factor TP53, which directly binds to the 3 ^′^UTR of miR‐34 mRNA [16]. However, miR‐34b/c does not appear to play a predominant role in regulating the TP53 gene, as one study found that overexpression of miR‐34b or miR‐34c, unlike overexpression of miR‐34a, had little effect on TP53 promoter activity and only weakly increased mRNA levels of TP53 transcriptional targets [17]. Therefore, the observed increase in TP53 levels in the present study was probably not a direct result of miR‐34b transfection into cells, but rather a consequence of increased BRD4 expression. A study by Zhou et al. previously discussed the interaction between TP53 and BRD4, revealing that BRD4 promotes mutant TP53 in TNBC. Thus, inhibition of BRD4 reduced mutant TP53 protein levels, leading to increased p21 expression and halted cell proliferation, whereas the upregulation of BRD4 expression elevated mutant TP53 levels in TNBC cells [14]. Based on this evidence, it can be inferred that the stimulatory effect of miR‐34 on mutant TP53 is likely an indirect effect and contributes to the increased BRD4 levels in MDA‐MB‐231 cells. Various studies have shown the reciprocal interaction between BRD4 and mutated TP53 in cancer cells. Specifically, according to the UALCAN cancer dataset report, breast cancer samples with mutant TP53 have higher BRD4 expression compared to those with nonmutant p53 [18]. Additionally, the expression of BRD4 in TNBC is highest among all three subclasses (luminal, HER2 positive, and triple negative) of breast cancer [19]. In esophageal squamous cell carcinoma (ESCC), specific mutant forms of TP53 (p53‐R172H) have been linked to increased metastasis through mechanisms involving BRD4. The interaction between mutant TP53 and BRD4 enhances the transcription of colony‐stimulating factor‐1 (CSF‐1), which promotes tumor cell invasion and metastasis [20]. All these previous reports confirm that the increase in BRD4 leads to an increase in TP53 in MDA‐MB‐231 cells, ultimately resulting in more aggressive behavior in them. However, this effect was not observed in MCF‐7 cells, belonging to the estrogen receptor positive (ER^+^) type of breast cancer. The distinct role and function of a common miR family in breast cancer subtypes has been reported before. Hassain et al. investigated the expression and role of the miR‐17–92 cluster in TNBC and ER^+^ breast cancer cells. They found that introducing miR‐17–92 reduced cell growth and invasion in ER^+^ breast cancer cells, while in contrast, miR‐17–92 expression actually increased growth and invasion in TNBC cells [21].

Bromodomain inhibitors including JQ1 represent a promising class of targeted therapies for breast cancer through disrupting the function of BET proteins (BRD2, BRD3, and BRD4) by preventing their binding to acetylated lysines on histones, leading to a transcriptional downregulation of key oncogenic genes driving tumor growth, cell survival, and metastasis [22]. A previous study by Lu et al. demonstrated that JQ1 treatment strongly inhibited the migration of MDA‐MB‐231 cells, even in the presence of TGF‐*β* as an inducer of epithelial–mesenchymal transition (EMT). Their findings revealed that JQ1 treatment decreased the expression of EMT‐promoting factors including fibronectin and Snail family transcriptional repressor (Snail) factors [23].

The results of the current study showed that JQ1 effectively inhibited BRD4 gene expression in both cell lines; however, no significant reduction was observed in BRD4 expression in MDA‐MB‐231 cells transfected with miR‐34b. Considering that BRD4 expression significantly increased in MDA‐MB‐231 cells following miR transfection, but remained unchanged in MCF‐7 cells, these findings suggest that JQ1 failed to reduce BRD4 expression in the transfected MDA cells. Given the crucial role of the BRD4 gene in cell migration, the effect of JQ1 on the migration rate of cells transfected with miR‐34b was evaluated. The results indicated that JQ1 exhibited a less inhibitory effect on migration in transfected MDA‐MB‐231 cells compared to transfected MCF‐7 cells, with transfected MCF‐7 cells showing almost no significant migration within 24 h. Meanwhile, treatment with JQ1 had no significant effect on inhibiting the migration of transfected MDA‐MB‐231 cells. This could be due to increased expression of BRD4 in transfected MDA‐MB‐231 cells. The inhibitory role of JQ1 on BRD4 causes it to act as a disconnecting agent on the BRD4/mutant TP53 axis and thus prevents the stimulating action of BRD4 on TP53, and this is how a previous study showed that JQ1 blocks BRD4’s ability to promote mutant TP53 gene expression. Consequently, treatment with JQ1 leads to decreases in mutant TP53 mRNA and protein levels. Along with cell proliferation, mutant TP53 increases the expression of EMT transcription factors such as Twist1 and Zeb1, thereby promoting metastasis [24]. Considering that following miR‐34b overexpression, both BRD4 and TP53 levels were elevated in MDA‐MB‐231 cells; presumably, JQ1 was not effective in blocking BRD4 activity and subsequently inhibiting cell migration. This finding warrants further investigation, particularly regarding the range of factors influencing migration, including the Jagged‐1 (JAG1)/Notch1 signaling pathway, which plays a role in the anticancer activities of both miR‐34b and BRD4. Among previous studies that could strengthen this idea is a study by Lee et al., which showed that miR‐34b acts as a tumor‐suppressing agent in breast cancer cells through suppression of JAG1 [25]. Also, regarding BRD4, a study showed that in TNBC, there is a direct relationship between BRD4 and JAG1, such that BRD4 overexpression leads to the upregulation of JAG1 mRNA and protein in MDA‐MB‐231 cells, but not in MCF‐7 cells, ultimately resulting in increased migration and invasion [26]. Considering these reports, the JAG1/Notch1 pathway may also contribute to the results obtained in the current study.

## 5. Conclusion

The current study indicates that miR‐34b likely has distinct roles in regulating cellular processes based on BRD4 and TP53 status in breast cancer cells. While combining miR‐34b with JQ1, a BRD4 inhibitor, could present a therapeutic option for breast cancer patients, this approach warrants further investigation, particularly in the context of TNBC, where the oncogenic role of miR‐34b must be carefully considered.

## Disclosure

All authors have read and agreed to the published version of the manuscript.

## Conflicts of Interest

The authors declare no conflicts of interest.

## Author Contributions

Conceptualization: M.A.I. and H.J.; methodology: M.A.I.; validation: M.A.I. and H.J.; formal analysis: M.A.I. and H.J.; investigation: M.A.I. and H.J.; resources: M.A.I.; data curation: H.J.; writing—original draft preparation: M.A.I. and H.J.; writing—review and editing: H.J.; supervision: H.J. and M.A.; project administration: H.J.

## Funding

No funding was received for this manuscript.

## Data Availability

Data are available upon request.
